# Characteristics of temporomandibular joint structures after mandibular condyle fractures revealed by magnetic resonance imaging

**DOI:** 10.1186/s40902-016-0066-0

**Published:** 2016-06-25

**Authors:** Bong Chul Kim, Yoon Chang Lee, Hyung Seok Cha, Sang-Hwy Lee

**Affiliations:** 1 Department of Oral and Maxillofacial Surgery, Daejeon Dental Hospital, Wonkwang University College of Dentistry, Daejeon, South Korea; 2 Department of Oral and Maxillofacial Surgery and Oral Science Research Center, College of Dentistry, Yonsei University, 50-1 Yonsei-ro, Seodaemun-gu Seoul, 120-752 South Korea

**Keywords:** Mandibular condyle, Mandibular fractures, Temporomandibular joint, Magnetic resonance imaging

## Abstract

**Background:**

This study aimed to evaluate the structural changes of temporomandibular joint immediately after condylar fractures with magnetic resonance imaging (MRI).

**Method:**

We evaluated 34 subjects of condylar fractures with MRI. The position, shape, and signal intensity of the condyle, disc, and retrodiscal tissue were analyzed with MR images.

**Results:**

Immediately after trauma, the disc was displaced with the fractured segment in almost all cases. And, the changes of signal intensity at the retrodiscal tissue were found but less related to the degree of fracture displacement. And, the high signals were observed almost at all fractured joint spaces and even at some contralateral joints.

**Conclusions:**

The displaced disc as well as the increased signal intensity of the joint space, condylar head, and retrodiscal tissue demands more attention to prevent the possible sequela of joint.

## Background

Although the condylar process of the mandible locates away from the direct traumatic insults, it is a structure with frequent facial traumatic injury. The forceful impact brings the fracture at this long thin anatomical structure by the transmission of the traumatic forces. The frequency of the condylar fracture ranges from 17.5 to 52 % [1–10], and it might be categorized as one of the controversial fractures in its diagnosis and management for facial bone fractures [10].

Meanwhile, the radiographic modalities, such as the transcranial temporomandibular joint (TMJ) view, panoramic view, and TMJ tomogram, have long been used for the diagnosis of the TMJ region. TMJ arthrogram has been added to its use with improved imaging quality for description of joint space and condylar shape. Finally, the arthroscope and the magnetic resonance imaging (MRI) have been introduced for more effective diagnosis of TMJ. Among all modalities, nowadays, MRI has become one of the most widely effective imaging modalities for TMJ. It can produce relatively accurate images on the shape and position of discs, characteristics of joint space, and its surrounding tissues.

It has been rarely reported about the nature of TMJ after the mandibular condyle fracture. This information is essential for the introduction of proper treatment, such as the selection of surgical or nonsurgical modality, the timing of treatment, and the necessity of the disc management [10]. Therefore, in this study, we took MRI images for the condylar fractures immediately after the traumatic injuries and analyzed them to evaluate the conditions of TMJ, which were injured and partly deformed by trauma with the fractured displacement of mandibular condyle. This allowed us to understand the changed nature of traumatic condyle and TMJ and the way of delivering our surgical protocol toward the healthy recovery of the structure.

## Methods

The subjects were selected for the patients with condylar fracture and for whom their MRIs were taken immediately after the traumatic injuries. Thirty-four subjects with MRIs were chosen, and they had 47 fractured joints of TMJ and 21 contralateral joints (total 68 joints). Table 1 shows characteristics of the studied subjects including the number of fractured joints, mean age, and average time after trauma. Table 2 represents the relationship between the position of fractured condylar segments on MRI and plain X-rays.

**Table 1:** Characteristics of the studied subjects and their joints

	Fractured	Contralateral	Total
TMJ	47	21	68
Average time after trauma	7 days		
Average age	29 years old

**Table 2:** Comparison of the fractured condylar segment position by MRI and panoramic X-rays

MRI plain X-rays	*N*	At eminence	In fossa				Normal
			Anterior	Anterior + down	Down	Posterior	
Dislocation	18	18 (38)					
Displacement	10	5 (11)			3 (6)		2 (4)
No displacement	15			1 (2)	3 (6)	6 (13)	5 (11)
Lateral displacement	2					2 (4)	
Chip fracture	2						2 (4)
Contralateral	21	1 (5)		1 (5)		11 (52)	8 (38)

( ): % in total fractured or contralateral joints

*p* < 0.01 (statistically significant for segmental position by MRI and fracture type by plain X-ray and chi-square test)

Several suggestions have been made for the classification of condylar fracture on the basis of anatomical position or relationship with the glenoid fossa [11, 12]. We wanted to adopt the classification by Lindahl [11], because of their rational and easy application. We classified the fractures of our subjects to be displacement or dislocation based on the findings of panoramic and Towne’s view.

MRI was taken for each subject immediately or as soon as possible after trauma, but before the initiation of any form of treatment with 1.5 Tesla Magnetom 63SP 4000 (Siemens medical system, Erlagen, Germany) using head surface coil. The condition was set as follows: the proton-weighted image (TR 2000, TE 15, FOV 150 cm, ACQ 1, thickness 3 mm, matrix 224 × 256) and T2-weighted image (TR 4000, TE 90, FOV 150 cm, ACQ 1, thickness 3 mm, matrix 240 × 256). The proton-weighted image of axial and sagittal plane and T2-weighted image of coronal plane were reconstructed independently. This work was approved by the local ethics committee of the Daejeon Dental Hospital, Wonkwang University College of Dentistry, Daejeon, Korea (IRB number: W1512/004-001) who waived the need for informed consent.

One radiologist and one of the authors (SHLee) observed and analyzed the shape, position, and signal intensity for the condylar head with its shape, the degree of displacement and shape of disc, and the joint cavity (cavum articulare) on MRI images.

We predicted that the degrees of displacements in the fractured condylar segments were closely related to the position and the shape of the disc. So, we first wanted to analyze the amount and position of the displacement which occurred to the fractured condylar segment rather than the simple location or level of condylar fractures. We placed it as the independent variable describing the proximal segment of the mandible. This was applied as long as the condylar segment was not small enough, such as in chip fracture. For the chip fracture of the condylar head, the main condylar part was used as a basis rather than to analyze the displaced chip fragment.

The amount of displacement of the fractured condylar segment was judged by the plain X-rays and categorized as the dislocation, displacement, or no displacement based on position of the relative position between condylar head and articular fossa according to Lindahl [11]. To evaluate disc position, disc in the closed-mouth position was classified into four compartments on the basis of the position of the posterior band of the disc in relation to the functional surface of the condyle following the criterion set by Murakami et al. [13]. Then, Hirata et al. modified this criterion and described disc position as superior, anterosuperior, anterior, and anteroinferior [14]. In case that the disc was displaced over the “anteroinferior” compartment, we allocated them “at eminence”. And, the results were statistically evaluated with chi-square test, and the significance level was confirmed on the basis of the *p* value 0.05.

## Results

Table 2 shows the positional distribution of the fractured condylar segment (on MRI) in relation to the degree of displacement of the segment (on plain X-rays). The dislocation group had 18 joints, the displacement group 19 joints, no displacements 15 joints, and the contralateral joints 21. When we analyze the correlation between the position of fractured condylar segments (on MRI) and the displacement of the condylar head (as dislocation and displacement on plain X-rays), the results were somewhat different from our expectation.

It showed that the degree of fracture displacement based on the plain X-rays was not completely matched with the final position of the fractured condylar segment on MRI; there were five cases from the displacement group (but not the dislocation group) which showed the condylar segments being completely luxated from glenoid fossa and/or even from articular eminence. They reached 50 % of displacement group, and the others of the remaining displacement group were found to be normal or inferiorly positioned (*N* = 2 and 3 each). For those with dislocation, the condyles were all located near articular eminence, away from fossa. And, some of the condylar segments from the group of no displacement (*N* = 6), the lateral displacement (*N* = 2) and contralateral side (*N* = 11) had their condylar segments being located at the more posterior position. All others were same as the displacement degree of the fractured condylar segment.

**Fig. 1:**
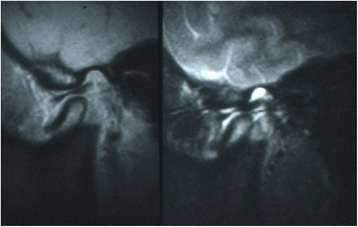
On proton density-weighted images in the closed-mouth position, the condylar head and disc show a downward displacement with normal condyle-disc relation and disc shape. On T2-weighted image in the closed-mouth position, high signal intensity is found in posterosuperior joint space. It is regarded as a hemarthrosis

**Fig. 2:**
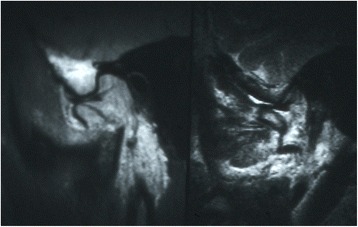
On proton density-weighted images in the closed-mouth position, the condylar head and disc show a dislocation that they are located near articular eminence, away from the fossa. Rather than normal condyle-disc relation, the condyle is found at the anterior, and disc shape appears normal

The analysis about the degree of disc displacement was followed, and they showed the similar results in general sense with the previous analysis about the condylar segment displacement (Figs. 1 and 2, Table 3). Among the cases in condylar dislocation group, only one was observed to be located within the glenoid fossa at the lower position, and all others were located at the area inferior to the articular eminence. So, the location of the disc in this group dictated exactly the displaced position of the condylar segment for all samples of the dislocation group except one. When it came to the displacement group, the degree of disc displacement was almost the same as that of the condylar segment; though some discs were moved further forward.

**Table 3:** The relationship between the position of the disc on MRI and the displacement of the condylar head on plain X-rays

MRI plain X-rays	*N*		Position of disc			
		At eminence	In fossa			Normal
			Anterior	Down	Anterior + down	
Dislocation	18	17 (36)			1 (2)	
Displacement	10	5 (11)		1 (2)	2 (4)	2 (4)
No displacement	15		3 (6)	2 (4)	2 (4)	8 (17)
Lateral displacement	2		2 (4)			
Chip fracture	2	1 (2)				1 (2)
Contralateral	21	1 (5)	4 (19)	4 (19)	1 (5)	11 (52)

( ): % in total fractured or contralateral joints

*p* < 0.01 (statistically significant for disc position and fracture, chi-square test)

When we evaluated no condylar displacement group, some discs (*N* = 6) were displaced to the anterior and inferior position within the fossa. And, six other cases of this group were found to have discs posteriorly positioned within the fossa, while three joints had the discs anteriorly displaced.

The lateral condylar displacement group had the discs all situated at the anterior region of the glenoid fossa. And, the chip fracture group showed that one disc was displaced to the inferior area of eminence along with the chip fragment, while another case was at the normal position within the fossa. In addition, the contralateral group had 11 joints (53 % of total contralateral cases) with the normal positioned discs, while other remaining samples were displaced variously to the anterior or inferior area of glenoid fossa or even to the inferior region of the eminence.

When it came to the analysis of the inter-relationship between discs and the condyle, the most of the cases (*N* = 13/18) of condylar dislocation group had the anterior-superior portion of condyle or load-bearing area being placed at the inferior area of the disc’s intermediate zone (Table 4). But, the displacement group had no condylar load-bearing area being positioned at the anterior region of intermediate zone, but most of them were located at the normal (*N* = 8) or posterior region (*N* = 2). The lateral dislocation group had all their two cases at the posterior region. And, the contralateral group showed 12 condyles located more posterior to the discs, while nine cases at the normal condyle-disc relation.

**Table 4:** The position of the condyle in relation to the disc on MRI

	Condyle position		
	Anterior to disc	Normal	Posterior to disc
Dislocation	13 (28)	5 (11)	
Displacement		8 (17)	2 (4)
No displacement		7 (15)	8 (17)
Lateral displacement			2 (4)
Chip fracture		1 (2)	1 (2)
Contralateral		9 (43)	12 (57)

( ): % in total fractured or contralateral joints

*p* < 0.01 (statistically significant for condyle position and fracture, chi-square test)

**Fig. 3:**
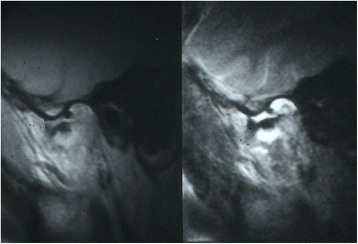
Sagittal proton density-weighted image shows a condylar head fracture. It also demonstrates that the disc is deformed and anteriorly displaced. Sagittal T2-weighted image demonstrates a disruption of the oblique line representing capsule. It is thought to be a capsular tear. Also, joint effusion and hemarthrosis are found in the superior and inferior joint space

When we analyzed the shape of the discs, they were generally normal (Fig. 3, Table 5). But, their shapes were different from the normal ones in seven cases (15 %) from the fracture side and one case (5 %) of contralateral side, respectively. Among those deformed discs from the fracture side, the dislocation group had four cases and the displacement group three, while no cases were found in the other groups (details were not shown in the table). In addition, we could find some tearing at the retrodiscal area from the fracture side (*N* = 6), and they were almost the same cases of the deformed discs.

**Table 5:** Shape of the disc and retrodiscal tissue at trauma on MRI

Side	Disc		Retrodiscal tissue	
	Deformed	Normal	Teared	Normal
Fractured	7 (15)	40 (85)	6 (13)	41 (87)
Contralateral	1 (5)	20 (95)		21 (100)

( ): % in total fractured or contralateral joints

*p* > 0.05 (statistically insignificant for disc/retrodiscal tissue and fracture, chi-square test)

The signal intensity of condylar area appeared to be normal in general, while two high signal intensities of T2-weighted image were found from no displacement group and one high case from the contralateral group, where almost no deviation of the condyle was found (Table 6). And, the same high signals of the retrodiscal tissue were found most frequently from the dislocation group with seven cases, while the displacement group with two cases, no displacement group with two cases, and even the contralateral group with two cases.

**Table 6:** The signal intensity of the condyle and retrodiscal tissue on MRI

	Condyle			Retrodiscal tissue		
	High	Low	Normal	High	Low	Normal
Dislocation			18 (38)	7 (15)		11 (23)
Displacement			10 (21)	2 (4)		8 (17)
No displacement	2 (4)		13 (28)	2 (4)		13 (28)
Lateral displacement			2 (4)			2 (4)
Chip fracture			2 (4)			2 (4)
Contralateral	1 (5)		20 (95)	2 (10)		19 (91)

( ): % in total fractured or contralateral joints

*p* > 0.05 (statistically insignificant for signals of disc/retrodiscal tissue and fracture, chi-square test)

The different signals were also found at the joint space on MR images. On fracture side, the superior and/or inferior joint space was observed to have higher signal intensity than normal at most of the fracture side T2-weighted images (Table 7). The high signal intensity was found at the superior joint space in 91 % of fractured joints (*N* = 43), while somewhat lower frequency at the inferior joint space (*N* = 34, 72 %). And, this high signal was also found at the contralateral side group, eight cases at the superior joint space, and nine at the inferior space.

**Table 7:** The signal intensity of the joint space after trauma on MRI

Side	Superior			Inferior		
	High	Low	Normal	High	Low	Normal
Fractured	43 (91)		4 (9)	34 (72)		13 (28)
Contralateral	8 (38)		13 (62)	9 (43)		12 (57)

( ): % in total fractured or contralateral joints

*p* < 0.05 (statistically significant, chi-square test)

The increased signal intensity at the condylar fossa or articular eminence was not found at the fractured or contralateral side, except only one case from the contralateral group (Table 8). In addition, the mastoid process also showed the high signal intensity at fractured groups (*N* = 8) as well as at the contralateral group (*N* = 1).

**Table 8:** The signal intensity of the fossa, eminence, and mastoid after trauma on MRI

	Fossa-eminence			Mastoid		
	High	Low	Flat	Normal	High	Normal
Fractured				47 (100)	8 (17)	39 (83)
Contralateral	1 (5)			20 (95)	1 (5)	20 (95)

( ): % in total fractured or contralateral joints

*p* > 0.05 (statistically not significant, chi-square test)

## Discussion

TMJ is a bilateral joint structure where both rotational and translational movement occurs. As mentioned previously, MRI can image the nature of condylar structure, a component of TMJ, more effectively than the plain X-ray or arthrogram does [15, 16]. Also, MRI is currently the most representative diagnostic method for the assessment of the soft tissue component of TMJ [17]. So, for the imaging TMJ structures, whether it is bony or soft tissue, MRI will be the best structure we can rely on today.

On the condylar fractures, the positional changes of the condylar head occur, in terms of the displacement or dislocation, and they in turn accompany inevitably the deformation or destruction of TMJ structure. And, this suggests the possible problems of disc position or structure and also destructional changes to joint capsule and/or surrounding ligaments. Christiansen et al. [18] investigated 43 patients with TMJ trauma using the CT and reported that the disc displacement was observed in 16 patients (37 %) while 15 patients (34 %) had the mandibular fracture. Raustia et al. [19] also examined 46 post-traumatic condylar fractures of 40 patients using the CT and plain X-rays after an average of 47 months and reported that the anterior disc displacement was found in six joints among 21 joints, which was far more than expected.

But, it would not be easy to expect their results to be reliable, since it was hard to gain an accurate understanding of structural changes for TMJ by CT imagings. Therefore, Jones et al. [20] and Goss et al. [21] evaluated, respectively, 14 and 20 patients of condylar fracture with arthroscope, and reported that discs were conserved intact in all cases, even though the considerable synovial ecchymosis was observed. These reports were, however, argued by subsequent studies using MRI. Sullivan et al. [22] and Takaku et al. [23] have reported that all the discs were displaced in the anterior/inner directions along with the fractured condyle, which was contrary to the findings from arthroscope-based studies.

This study aimed at presenting the possible disc displacement with accurate MRI imaging. We could find differences between the displacement degree of fractured fragment (on plain X-rays) and displacement of fractured proximal segment of condyle (on MRI) (Table 2). This probably came from the lack of information obtained from plain X-rays with the limited disclosure of the degree of fractured fragment displacement. It also raised the possibility of more distant segmental displacement and also the need of more careful diagnosis and treatment planning when we rely on the plain X-rays. The displacement pattern of the TM disc seemed to be similar situation as that of condylar segment (Table 3). And, our observation about the positional relationship between the condyle and disc indicated that the general position of the disc was near the condylar segment, especially when we disregard the minor difference of anterior or posterior discal position (Table 4). Dwivedi et al. [17] reported a significant relationship between the extent of condylar fracture and the degree of impairment to the disc. But, it also indicated that the final positional relationship between the displaced condylar and discal structure were not always identical. The number of condylar position “near eminence” from the dislocation and displacement group consisted of 23 cases out of 28 cases (Table 2). And, the number of disc positions near the eminence was 22 cases (Table 3). In addition, the condylar segments from the non-displacement group had six cases of posterior position within the glenoid fossa, while none from the same group had the posterior disc positions. All these findings indicated that the position of the disc went along with the condylar fracture segment, but with more anterior positional tendency.

Changes of the disc shape were found in seven cases on the fracture side and one case on the contralateral side (Table 5). In most cases, disc shapes were changed to have the anterior bending, which is typically different in shape from that of TMD with disc displacement with/without reduction. This morphological change seemed to be related to the traumatic injury, but hard to explain due to the lack of information. According to Dwivedi et al. [17], the higher condylar fracture tends to cause the greater injury to retrodiscal tissue, and to cause the lower capsular tears with the minimally displaced condylar fracture.

The morphological changes of the retrodiscal tissue were also thought to be caused by the traumatic injury, to indicate the tearing by the anterior dislocating condylar segment along with the disc. We could find this kind of injury to the retrodiscal tissue in six cases (13 %) at the condylar fractured sides (Table 5), while 39 to 50 % by Sullivan et al. [22] and Takaku et al. [23]. They regarded the retrodiscal tissue as highly susceptible to damage associated with condylar fractures.

However, the signal changes of retrodiscal tissue were found in seven cases from dislocation group, two cases from displacement and non-displacement group, and even two cases from contralateral side (Table 6). Thus, it seemed not to be highly related with the degree of displacement. And, this signal intensity change was found to be localized at the area immediately posterior to the disc, without any low signal intensity. Thus, this signal changes seemed to be correlated with the high chance of strong impact onto the retrodiscal tissue by direct trauma or indirect to the displaced condylar segment. In the same context, Sano et al. [24] reported that the high signal intensity of the retrodiscal tissue on T2-weighted images could be related closely to the severe joint pain. And, Takaku et al. [23] has reported that the existence of abnormal signal intensity of retrodiscal tissue after trauma might suggest the presence of trauma-induced inflammation.

The occurrence of high signals at both the condylar segment and retrodiscal tissue were found in three cases, and they were all minimal or undisplaced fractures. This finding shows the possible great traumatic injury to the condylar head and its adjacent tissues even if they are not accompanied by the fracture. And, it also raises the possibility of the higher traumatic injury onto the non-fracture side or minimal displaced fracture. Thus, we need to pay the same attention to the non-fracture side as to the fractured condyle.

Regarding the signal intensity (on T2-weighted images) of the joint space, they were found in 91 % (43 joints) of fracture side and 38 % (eight joints) of contralateral side for the superior joint cavity, and 72 % (34 joints) of fracture side and 43 % (nine joints) of contralateral side for the inferior space (Table 7). It could be diagnosed as the hemarthrosis, which might be mixed with bleeding and synovial fluid within the joint cavity.

In arthroscope-based studies, Jones et al. [20] found the hemarthrosis joints with condylar fractures in 13 joints out of 15 cases, and Goss et al. [21] observed it from 82 % cases when observed within 5 days. And, Takaku et al. [23] observed the high signal intensity from all of the 12 joints on MR images and found hemarthrosis on six joints during the surgery. Gerhard et al. [25] also found the significant relationship between the degree of condylar injury and hemarthrosis findings on MRI. These authors all proposed the hemarthrosis when the capsule or retrodiscal tissue was damaged. And, their significances are definitely related to the possible adhesion or ankylosis of the joints in case we fail to manage them properly.

## Conclusions

We evaluated the changes of temporomandibular joint immediately after condylar fractures with MRI for 34 subjects. All the components of the temporomandibular joint were examined, including the position, shape, and signal intensity of the condyle, disc, and retrodiscal tissue.

1. Some of the condylar fractures on plain X-rays were presented differently on MRI in terms of the displacement degree.
2. The temporomandibular disc was displaced along with the fractured segment in almost all cases, and this was the most prominent in the condylar dislocation group.
3. The position of the disc generally matched with the position of the condylar segment.
4. The increased signal intensity was prominent at the retrodiscal tissue, but this was not directly related to the degree of fracture displacement.
5. The morphological change of disc was infrequently found in seven cases at the fracture side and one case on the contralateral side, but not for the ruptured disc.
6. The high signals within the joint space, suggesting the hemarthrosis, was observed almost in 92 % of fractured joints and in 38 % of contralateral joints.

The displaced disc as well as the increased signal intensity of the joint space, condylar head, and retrodiscal tissue demand more attention from surgeons to prevent the possible sequela of joint trauma.
